# Severely Elevated Blood Pressure and Early Mortality in Children with Traumatic Brain Injuries: The Neglected End of the Spectrum

**DOI:** 10.5811/westjem.2018.2.36404

**Published:** 2018-04-05

**Authors:** M. Austin Johnson, Matthew A. Borgman, Jeremy W. Cannon, Nathan Kuppermann, Lucas P. Neff

**Affiliations:** * University of California Davis Medical Center, Department of Emergency Medicine, Sacramento, California; † Brooke Army Medical Center, Department of Pediatrics, Ft Sam Houston, Texas; ‡ Uniformed Services University of the Health Sciences, Department of Pediatrics, Bethesda, Maryland; § Perelman School of Medicine at the University of Pennsylvania, Department of Surgery, Philadelphia, Pennsylvania; ¶ David Grant USAF Medical Center, Travis Air Force Base, Department of General Surgery, Fairfield, California; || University of California Davis Medical Center, Department of Surgery, Sacramento, California; # Uniformed Services University of the Health Sciences, Department of Surgery, Bethesda, Maryland

## Abstract

**Introduction:**

In adults with traumatic brain injuries (TBI), hypotension and hypertension at presentation are associated with mortality. The effect of age-adjusted blood pressure in children with TBI has been insufficiently studied. We sought to determine if age-adjusted hypertension in children with severe TBI is associated with mortality.

**Methods:**

This was a retrospective analysis of the Department of Defense Trauma Registry (DoDTR) between 2001 and 2013. We included for analysis patients <18 years with severe TBI defined as Abbreviated Injury Severity (AIS) scores of the head ≥3. We defined hypertension as moderate for systolic blood pressures (SBP) between the 95^th^ and 99^th^ percentile for age and gender and severe if greater than the 99th percentile. Hypotension was defined as SBP <90 mmHg for children >10 years or < 70mmHg + (2 × age) for children ≤10 years. We performed multivariable logistic regression and Cox regression to determine if BP categories were associated with mortality.

**Results:**

Of 4,990 children included in the DoDTR, 740 met criteria for analysis. Fifty patients (6.8%) were hypotensive upon arrival to the ED, 385 (52.0%) were normotensive, 115 (15.5%) had moderate hypertension, and 190 (25.7%) had severe hypertension. When compared to normotensive patients, moderate and severe hypertension patients had similar Injury Severity Scores, similar AIS head scores, and similar frequencies of neurosurgical procedures. Multivariable logistic regression demonstrated that hypotension (odd ratio [OR] 2.85, 95 confidence interval [CI] 1.26–6.47) and severe hypertension (OR 2.58, 95 CI 1.32–5.03) were associated with increased 24-hour mortality. Neither hypotension (Hazard ratio (HR) 1.52, 95 CI 0.74–3.11) nor severe hypertension (HR 1.65, 95 CI 0.65–2.30) was associated with time to mortality.

**Conclusion:**

Pediatric age-adjusted hypertension is frequent after severe TBI. Severe hypertension is strongly associated with 24-hour mortality. Pediatric age-adjusted blood pressure needs to be further evaluated as a critical marker of early mortality.

## INTRODUCTION

Within the United States, an estimated 50,000 children are hospitalized and 3,000 die each year following traumatic brain injury (TBI).^1^ TBI accounts for almost one-half of all deaths in children older than one year of age and is a leading cause of lost productive life-years in the U.S. Increased recognition of TBI in children as a major cause of childhood morbidity and mortality has led to advances in injury prevention and emphasizes the need for new therapies to improve long-term outcomes.^1^–^3^ Despite this, substantial knowledge gaps remain in the early management of pediatric patients with TBIs.

A foundational aspect in the management of severe TBIs is optimization of systemic hemodynamics, as both hypotension and hypoxia have been shown to be associated with adverse outcomes.^4^–^6^ Cerebral perfusion is principally determined by the systemic blood pressure (BP) and intracranial pressure (ICP). Maximizing cerebral perfusion pressure through reductions in ICP and elevations in mean arterial pressure (MAP) are the basis for current treatment algorithms for adults with TBIs.^5^–^7^ After restoration of euvolemia by volume administration, vasopressors are used to increase MAP to maintain a cerebral perfusion pressure of at least 40 mmHg.^8^–^10^ It is unclear whether hypertension or “supernormal” BP is of any benefit, or potentially harmful, in pediatric patients.

Although hypertension during the first 72 hours after injury has been associated with improved outcomes in children with TBIs,^11^ emerging evidence in adult TBIs has identified an association between early episodes of hypertension and increased mortality.^12^–^14^ Elevated BP can lead to breakdown of the blood brain barrier and increase cerebral edema through hydrostatic forces.^15^ Although vasopressors are used at times to maximize cerebral perfusion, cerebral edema coupled with exogenous vasopressors can lead to a reduction in blood flow to at-risk brain regions that were initially non-ischemic.^16^ This suggests a complex relationship between systemic BP, particularly at extremes, and TBI outcomes.

We hypothesized that age-adjusted BP elevation is associated with worse outcomes in hospitalized children with TBIs. We analyzed the Department of Defense Trauma Registry (DoDTR) that contained data from 2001 to 2013 to describe associations between emergency department (ED) BP and outcomes in children with TBIs cared for at U.S. military hospitals in Iraq and Afghanistan.

## MATERIALS and METHODS

### Study Design, Setting, Population

This study was initiated and conducted under a protocol approved by the San Antonio Military Medical Center Institutional Review Board. This was a retrospective study of patients <18 years old entered into the DoDTR from 2001 to 2013 in Iraq and Afghanistan. This registry has been described in detail in prior publications.^17^–^18^ Briefly, the registry is a prospectively collected dataset of all military and civilian patients from military conflicts in which the U.S. participated. While in the theatre of war, trained nurses abstracted patient data into the DoDTR with no knowledge of future studies. There were no patient records available to obtain any further information.

**Population Health Research Capsule**

What do we already know about this issue?

In children with traumatic brain injuries, hypotension is associated with increased mortality. No studies have described an association between hypertension and mortality in these children.

What was the research question?

Among children with severe traumatic brain injuries (TBIs), is age-adjusted hypertension associated with mortality?

What was the major finding of the study?

Age-adjusted hypertension is common and strongly associated with 24-hour mortality among children with severe TBIs.

How does this improve population health?

Our findings suggest that age-adjusted hypertension is equally predictive of morality as hypotension in these children. Future work is needed to understand the mechanisms behind this finding.

For this study, we excluded patients with non-traumatic mechanisms of injury (drowning and asphyxiation), primary thermal injuries, and with an absent pulse or missing BP measurement. We used AIS scores to define severity of TBI to overcome any potential language barriers that may have confounded arrival Glasgow Coma Scale (GCS) scores, and to allow for the identification of a comparison group of patients with isolated thoracic and abdominal trauma. Patients were considered to have a severe isolated TBI if their Abbreviated Injury Scale (AIS) of head, neck, and cervical spine was ≥3 and all remaining AIS scores were <3. We identified a comparison group of children with non-TBI trauma as having an AIS-head <3 with AIS of the chest, abdomen, or pelvic region ≥3.

### Variables Collected and Outcome Measures

The primary outcome measures were 24-hour mortality and mortality prior to discharge. Demographic and physiologic data elements collected from the registry included age, sex, weight, arrival BP, heart rate, oxygen saturation, and temperature. Injury mechanism, the injury severity score, AIS scores, and the GCS score were collected as markers of injury severity. We also collected total resuscitation fluids administered, ED intubation, neurosurgical interventions, total number of hospital days, number of days of mechanical ventilation, intensive care unit (ICU) length of stay, 24-hour mortality, and survival to hospital discharge. Only the arrival BP is included in the DoDTR.

We generated age- and gender-adjusted BPs using the American Heart Association Guidelines for the Diagnosis of Pediatric Hypertension.^19^ The DoDTR dataset does not include height as an independent variable; therefore, we used the 50^th^ percentile for height for a given age and gender based on World Health Organization (WHO) child growth standards. Moderate hypertension was defined as the 95^th^ to 99^th^ percentile for age and sex and severe hypertension was defined as greater than the 99^th^ percentile. Hypotension was defined as a systolic blood pressure (SBP) <90mmHg for children older than 10 years, or less then 70mmHg + (2 × age) for children 10 years and younger. We defined a neurosurgical procedure as any procedure that required access to the cranium.^20^ Age-adjusted rates of bradycardia were calculated using Pediatric Advanced Life Support definitions.^21^

### Data Management and Statistical Analysis

We transferred all data into STATA version 12.0 (College Station, TX) for statistical analyses. All frequency data are presented as prevalence estimates with 95% confidence intervals (CI). Normally distributed continuous data were reported as mean with standard deviations (SD), and ordinal or non-normally distributed continuous data were described with medians with interquartile ranges. We performed bivariable analysis of categorical variables using the χ^2^ test, and we analyzed continuous variables using Student’s-t test, or one-way analysis of variance with Bonferroni correction, as appropriate. We performed multivariable logistic regression to estimate associations between demographics, arrival BP, and 24-hour survival, adjusting for other variables and potential confounders. We used a Cox-proportional hazards model to determine the association of BP with in-hospital mortality, using a time unit of days. Individual characteristics considered for the model were age, sex, mechanism of injury, ED GCS score, arrival oxygen saturation, arrival BP category, ISS, ED intubation, neurosurgical intervention, and whether mechanical ventilation was required. In our regression models, we used stepwise variable inclusion; however, we forced patient demographics and injury mechanisms into the final models.

To minimize bias and preserve study power, we used multiple imputation for missing values (STATA 12.0, College Station, TX). Arrival oxygen saturation was missing for 21.2% of patients and arrival GCS scores were missing in 7.6%. To perform the multiple imputation, we used a multivariable normal model to derive 10 datasets. Included in the model were the following variables: age, gender, AIS-head, ISS, arrival oxygen saturation, arrival SBP, ED intubation, volume of blood product transfused, neurosurgical procedure, days on ventilator, ICU days, total hospital days, death within the first 24 hours, and in-hospital mortality. We performed a sensitivity analysis using only complete cases to examine the assumptions of our imputation on arrival GCS and mortality.

## RESULTS

### Characteristics of Study Population

**Figure 1:**
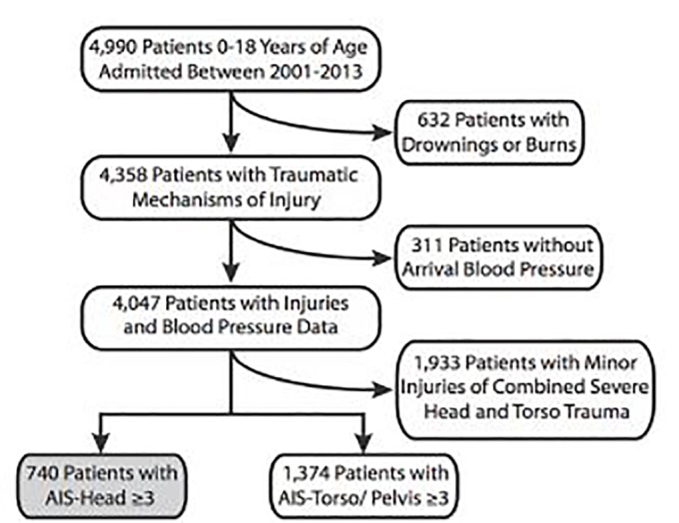
Study population of pediatric patients with traumatic brain injury. *AIS*, Abbreviated Injury Severity.

During the study period, 4,990 patients <18 years were included in the DoDTR. Of these, 632 patients (12.7%) were excluded for drowning or thermal burns, and 311 (6.2%) were excluded for missing SBP. Of the remaining 4,047 patients, 1,933 (47.8%) either had minor head injuries or had severe torso and pelvic injuries associated with their TBIs and were excluded from analysis. This left 740 patients (18.3%) with severe, isolated TBIs as defined by AIS, and 1,374 patients (34.0%) with isolated torso and pelvic injuries without an associated severe TBI (Figure 1).

### Main Results

Patients 0–2 years old had the highest incidence of severe hypertension (17, 50.0%) and patients 14–18 years old had the highest incidence of normotension (64, 65.3%) upon arrival to the ED (Table 1). There were no significant differences in rates of bradycardia across all BP categories. Patients who were hypotensive upon arrival to the ED had lower GCS scores, higher ISS, higher AIS-head, and were intubated more often than those who were not hypotensive on arrival (Table 1). There were no significant differences in ED GCS, ISS, AIS-head, or ED intubation between normotensive, moderate hypertensive, and severely hypertensive patients on post-hoc comparisons (Table 1).

**Figure 2:**
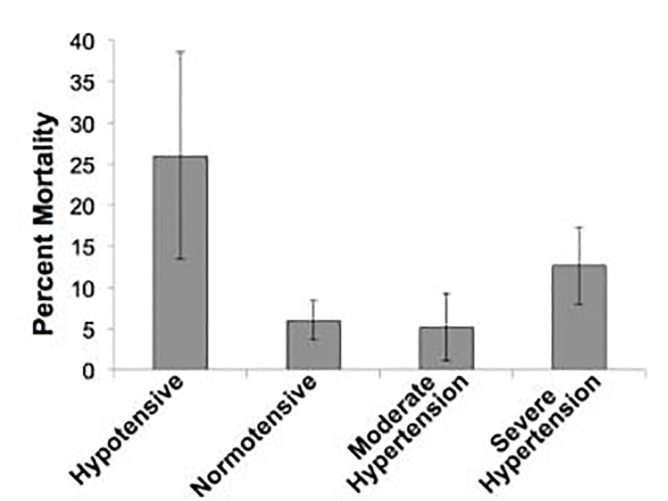
24-hour mortality by blood pressure category (%, 95CI).

Evaluation of 24-hour mortality and in-hospital mortality in an unadjusted analysis demonstrated a U-shaped distribution with hypotensive patients having the highest 24-hour and in-hospital mortality followed by patients with severe hypertension (Table 1, Figure 2).

**Figure 3:**
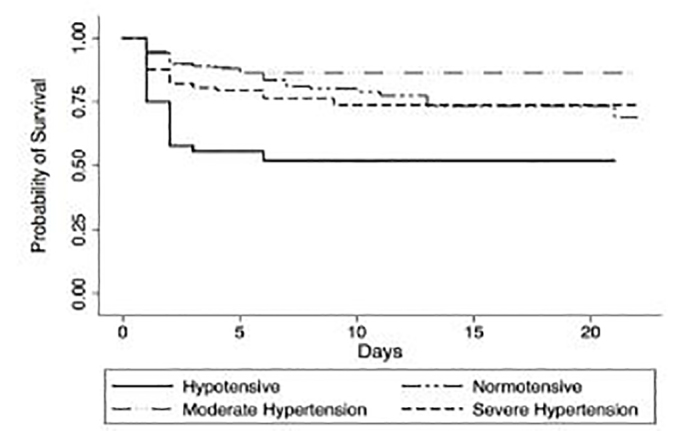
Kaplan Meier curve by blood pressure category.

Figure 3 demonstrates a Kaplan-Meier survival curve for each BP group. When compared to normotensive patients and patients with moderate hypertension, patients with severe hypertension had increased 24-hour mortality, which plateaued to mirror normotensive patients by 14 days. To determine if BP categories were associated with early mortality, we performed a multivariable logistic regression analysis to adjust for demographics and injury severity. Hypotension and severe hypertension were associated with increased 24-hour mortality (Table 2). A sub-analysis of patients presenting with a GCS ≤8 also demonstrated a U-shaped association between presenting BP and 24-hour mortality (Supplemental Table 1). In a Cox regression of in-hospital mortality adjusted for demographics and injury severity, hypotension and severe hypertension were associated with mortality, but once deaths within the first 24 hours were removed, arrival BP categories were no longer associated with in-hospital mortality (Table 3). A sensitivity analysis using complete cases demonstrated similar associations between BP categories and 24-hour mortality (Supplemental Table 2).

Finally, to determine if the association between hypertension and mortality was specific to patients with TBIs, we identified a second cohort of patients with isolated severe torso and abdominal trauma. In multivariable logistic regression analyses and Cox regression analysis severe hypertension was not associated with 24-hour mortality (Supplemental Table 3) or overall mortality (Supplemental Table 4).

## DISCUSSION

The present study demonstrates that age-adjusted BP greater than the 99^th^ percentile is common in children with severe TBIs, affecting approximately one-quarter of patients. Furthermore, BP greater than the 99^th^ percentile in children with isolated severe head injuries is associated with 24-hour mortality, and neither hypotension nor severe hypertension is associated with in-hospital mortality after accounting for early deaths. The strength of this association coupled with conflicting reports on the consequences of hypertension in pediatric TBI, reinforces the importance of continued investigation into the causes and effects of hypertension after TBI.

Analyses of hemodynamic parameters in critically injured children present substantial challenges due to the wide variation in age-adjusted normal BPs. For example, the upper limit of normal BP varies from 110 mmHg in a one-year-old female to 143 mmHg in a 17-year-old male.^19^ The need to account for differences in physiology based on age is essential in pediatric outcomes research. Prior pediatric TBI studies have demonstrated that age-adjusted hypotension, as compared to a fixed BP cutoff, is associated with worse outcomes,^6^,^22^ and in this manuscript we reported a rate of pediatric hypertension that is higher than rates reported in adult patients suffering from TBIs. The International Mission for Prognosis and Analysis of Clinical Trials in TBI Study, which combined results from nine large, randomized, controlled trials of adult patients with TBIs, reported that the incidence of hypertension varies from 10.3–36.5% with an average of 22%.^13^ By comparison, in the current pediatric cohort, the overall incidence of age-adjusted hypertension upon arrival to the ED was much higher.

Traditionally, children are thought to have a well-preserved ability to maintain vascular tone and BP even in the setting of early shock. It may be that these same cardiovascular mechanisms produce an exaggerated BP response to TBI, resulting in a high frequency of age-adjusted hypertension after TBI. If this effect is age-specific, with infants more greatly affected than older children, it would further explain the age-dependent frequency of severe hypertension that we observed. In our study, infants constituted the largest percentage of patients in both hypertension groups. Although one would assume that this is a protective mechanism to preserve cerebral blood flow, the etiology of the association with higher mortality is not clear and requires further study.

Within the field of adult neurotrauma, there is continued debate surrounding hypertension and TBI. Prior research has demonstrated that once a patient has been stabilized in the ICU, maximizing cerebral perfusion pressure with permissive hypertension or induced hypertension results in improved outcomes.^8^,^16^,^23^–^25^ Emerging evidence challenges this conventional wisdom. A large retrospective analysis of the National Trauma Data Bank demonstrated an increase in mortality with ED SBPs ≥140 mmHg on adjusted analysis.^12^ Similar studies using the European Trauma Database as well as the IMPACT dataset demonstrated an association between hypertension and mortality on bivariable analysis; however, this association was largely mitigated after adjusting for demographic and injury characteristics.^13^,^14^ Our findings are consistent with those of Zafar et al., which suggest an underlying pathology of early systemic BP after TBI that may be under-appreciated.

Changes in cerebral perfusion are thought to follow a distinct time course after TBI; an initial period of hypoperfusion during the first 6–12 hours is followed by a period of hyperemia, and finally a phase of hypoperfusion returns characterized by vasospasm and recurrent ischemia.^26^,^27^ This pattern of cerebral blood flow suggests that early episodes of hypertension could be neuroprotective by increasing cerebral perfusion pressure and overcoming periods of low flow. While characterization of cerebral blood flow and cerebral perfusion often occurs in the ICU setting after patients have been stabilized and may have undergone neurosurgical intervention, the BP measurements we report were recorded upon ED arrival, and likely represent an earlier phase of injury not described in previous studies.

The consequences of hypertension at this early time point may be different than hypertension several hours later after clot stabilization and the onset of increased ICP. Early hypertension may destabilize developing blood clots, increase vasogenic edema, and lead to increased ICP, while late elevations in SBP may maximize perfusion in the setting of increased ICP. Prospective trials with intensive, continuous SBP and ICP monitoring will be required to fully understand the role of BP early after TBI.

One of the only other studies to specifically investigate the role of hypertension in pediatric neurotrauma outcomes demonstrated that hypertension in the first 72 hours following severe TBIs (with GCS scores ≤8) was associated with improved outcomes.^11^ In that particular study, a non-age adjusted SBP of 135 mmHg was associated with an 18.8-fold increase in survival.^11^ A BP threshold of 135 mmHg is greater than the 99^th^ percentile for boys younger than 14 years and girls younger than 16 years,^19^ and stands in contrast to the current study’s findings. To limit ambiguity in the current study, patients with isolated and predominant head injuries were identified in order to reduce confounding of the hemodynamic effects and outcomes resulting from substantial non-cranial injuries. The inclusion of severe thoracic and abdominal trauma within the patient population studied by previous investigators may partially explain the improved outcomes in patients with hypertension. These discordant findings between studies emphasize the need for prospective analysis of patient hemodynamics and outcomes after TBIs in children.

Similar to other studies, we have demonstrated a strong association between hypotension on ED arrival and mortality.^4^,^6^,^12^–^14^ This patient population differed significantly from the other populations in several critical ways. First, the hypotensive patients had higher ISS, higher incidence of endotracheal intubation while in the ED, as well as lower arrival GCS scores. In general, this was a sicker patient population. The hypotensive patients also had a non-statistically significant higher frequency of penetrating trauma as the cause of injury when compared to the other groups, and in total these differences were associated with worse outcomes across all measured parameters including time on the ventilator, time in the ICU, and mortality. What is surprising is that once injury severity, GCS, and the need for emergent intubation were included in the regression models, both hypotension and severe hypertension had similar magnitudes of effect on mortality. Although the mechanisms of hypotension on poor outcomes have been linked to poor perfusion and tissue ischemia, further work is needed to understand potential mechanisms to explain the effect of hypertension on TBI.

The lack of radiographic analysis within the trauma database prevented estimation of the size of brain injury or the rate of cerebral herniation that may have accompanied hypertension. Rates of bradycardia, a proxy for cerebral herniation in the setting of hypertension, were not significantly different across all BP categories, suggesting hypertension was not independently associated with bradycardia and was not the result of cerebral herniation. Furthermore, inclusion of bradycardia within the regression models was not associated with increased mortality and did not change the point estimates for the severe hypertension group. Consequently, it was not included in the final regression models.

## LIMITATIONS

This study is subject to several limitations. In addition to the inherent issues of its retrospective design, the study patient population and the types of medical facilities within Iraq and Afghanistan differ from civilian medical centers and may limit the generalizability of our findings. Furthermore, due to a lack of published pediatric BP normal ranges in Iraq and Afghanistan, we had to use BP norms derived from U.S. pediatric BP to define BPs as moderate and severe hypertension. The DoDTR also lacks height as a recorded variable; therefore, we used the 50^th^ percentile for age based on WHO standards. Differences in normal rates of childhood heights in Afghanistan may have led to bias within the dataset. However, if bias was introduced based on smaller heights of Afghani children, it would bias more children into the moderate and severe hypertension groups.

The prehospital phase of care for patients within the current study was likely longer and the resuscitation during transport may not have been as intensive as practiced within the U.S. Although all military treatment facilities are equipped with both adult and pediatric BP cuffs, from this dataset we were unable to determine if the pediatric equipment was used for BP pressure reading. In addition, the DoDTR only includes a single BP reading taken upon arrival, which limited any ability to correlate changes in BP with outcomes.

Furthermore, it is unknown what level of resources was available to each individual patient. Resources were often limited based on type of facility as well as the number of other casualties, greatly limiting our ability to fully evaluate the frequency of neurosurgical interventions in each BP category. In addition, the DoDTR could not provide an accurate description of long-term outcomes or follow-up for host national patients. We have attempted to correlate early hypertension with cerebral herniation and increased ICP through the presence of bradycardia. The lack of intracranial monitoring and of sufficient available ICP data for complete evaluation, however, prevents adjustment for increased ICP. Limitations not withstanding, this study demonstrates important associations between initial arrival hemodynamics and short-term outcomes, which necessitates further investigation.

## CONCLUSION

In conclusion, we have demonstrated that in children with severe TBIs, marked age-adjusted hypertension is common and associated with early mortality. Early systemic hemodynamics after pediatric TBI requires further analysis to determine optimal resuscitation strategies.

## Supplementary Information









## Figures and Tables

**Table 1:** Patient demographics and injury characteristics stratified by blood pressure group.

	Hypotensive (N=50)		Normotensive (N=385)		Moderate hypertension (N=115)		Severe hypertension (N=190)		P value
Age (years) (N,%)									<0.01
0–2	1	2.9	15	44.1	1	2.9	17	50.0	
2–5	8	6.0	56	42.1	24	18.1	45	33.8	
5–10	20	7.1	138	48.9	45	16.0	79	28.0	
10–14	16	8.3	112	58.0	26	13.5	39	20.2	
14–18	5	5.1	64	65.3	19	19.4	10	10.2	
Sex (N,%)									0.81
Male	36	72.0	288	74.8	84	73.0	135	71.1	
Female	14	28.0	97	25.2	31	27.0	55	28.9	
Mechanism (N,%)									0.15
Blast	18	36.0	125	32.5	38	33.0	68	35.8	
Blunt	12	24.0	162	42.1	52	45.2	75	39.5	
Penetrating	20	40.0	98	25.5	25	21.7	47	24.7	
ED GCS (median, IQR)	3	3,6	9	3,9	9	3,15	9	3,15	<0.01
Arrival O_2_ saturation (mean, 95% CI)	95.1	91.5–98.8	97.4	96.5–98.3	98.1	97.1–99.0	97.1	96.0–98.3	0.23
Bradycardic in ED (N,%)	1	2.0	6	1.6	1	0.9	8	4.2	0.15
ISS (mean, 95% CI)	21.6	18.9–24.7	17.3	16.3–18.3	15.9	14.6–17.2	16.4	15.3–17.5	<0.01
AIS head (mean, 95% CI)	4.3	4.0–4.5	3.7	3.6–3.8	3.6	3.5–3.8	3.7	3.6–3.8	0.83
ED intubation (N,%)	33	66.0	148	38.4	36	31.3	58	30.5	<0.01
Neurosurgical procedure (N,%)	18	36.0	136	35.3	41	35.7	68	35.8	0.99
Ventilator days (median, IQR)	1.5	1,3	1	0,2	1	0,2	1	0,2	<0.01
ICU days (median, IQR)	2	1,5	2	1,4	2	1,4	2	1,4	<0.01
Hospital days (median, IQR)	2.5	1,6	3	2,7	3	1,6	3	1,6	<0.01
Died in 1st 24 hours (N,%)	13	26.0	23	6.0	6	5.2	24	12.6	<0.01
Died in hospital (N,%)	23	46.0	57	14.8	12	10.4	38	20.0	<0.01

*ED*, emergency department; *GCS*, Glasgow Coma Scale; *IQR*, interquartile range; *CI*, confidence interval; *ISS*, injury severity score; *AIS*, Abbreviated Injury Severity; *ICU*, intensive care unit.

**Table 2:** Multivariable logistic regression of mortality within first 24 hours.

	Odds ratio	95% CI	P
Age	1.0	0.93–1.07	1.0
Penetrating injury	0.98	0.53–1.81	0.95
ED GCS	0.72	0.65–0.80	<0.01
AIS head	1.69	1.21–2.36	<0.01
ED intubation	0.28	0.15–0.54	<0.01
Blood pressure			
Hypotensive	2.85	1.26–6.47	0.01
Normotensive		Reference	
95th-99th percentile	0.89	0.33–2.40	0.81
>99th percentile	2.58	1.32–5.03	<0.01

*CI*, confidence interval; *GCS*, Glasgow Coma Scale; *AIS*, Abbreviated Injury Scale; *ED*, emergency department

**Table 3:** Cox regression analysis of in-hospital mortality.

	All patients			Excluding 24 hour deaths		
		
	Hazard ratio	95% CI	P value	Hazard ratio	95% CI	P value
Age	1.00	0.96–1.05	0.77	1.00	0.94–1.06	0.97
Penetrating injury	1.14	0.78–1.65	0.50	1.25	0.74–2.11	0.40
ED GCS	0.79	0.74–0.84	<0.01	0.82	0.75–0.91	<0.01
AIS head	1.56	1.26–1.93	<0.01	1.62	1.19–2.20	<0.01
ED intubation	0.57	0.37–0.86	<0.01	0.90	0.46–1.74	0.75
Blood pressure						
Hypotensive	1.82	1.10–3.01	0.02	1.52	0.74–3.11	0.26
Normotensive	Reference					
95th–99th percentile	0.84	0.44–1.58	0.58	0.76	0.32–1.84	0.55
>99th percentile	1.65	1.09–2.52	0.02	1.22	0.65–2.30	0.54

*CI*, confidence interval; *GCS*, Glasgow Coma Scale; *AIS*, Abbreviated Injury Scale; *ED*, emergency department.
