# Supplementary material for: Severely Elevated Blood Pressure and Early Mortality in Children with Traumatic Brain Injuries: The Neglected End of the Spectrum
Source: West J Emerg Med. 2018 Apr 5;19(3):452–9. doi: 10.5811/westjem.2018.2.36404 (PMC5942007; doi:10.5811/westjem.2018.2.36404)
Supplement: Supplementary file 1 [file wjem-19-452-s001.docx]

**Supplemental Table 1: 24-Hour Mortality of Patients with GCS Scores ≤8**

|  | **Odds Ratio** | **95% CI** | **P** |
| --- | --- | --- | --- |
| **Age** | 1.0 | 0.93-1.08 | 0.9 |
| **Penetrating** | 1.03 | 0.55-1.95 | 0.93 |
| **ED GCS** | 0.74 | 0.59-0.93 | 0.01 |
| **AIS Head** | 1.78 | 1.26-2.53 | <0.01 |
| **ED Intubation** | 0.31 | 0.16-0.60 | <0.01 |
| **Blood pressure** |  | | |
| **Hypotensive** | 2.92 | 1.26-6.78 | 0.01 |
| **Normotensive** | Reference | | |
| **95^th^-99^th^ Percentile** | 0.89 | 0.33-2.40 | 0.81 |
| **>99^th^ Percentile** | 2.59 | 1.25-5.37 | 0.01 |
