# Supplementary material for: Severely Elevated Blood Pressure and Early Mortality in Children with Traumatic Brain Injuries: The Neglected End of the Spectrum
Source: West J Emerg Med. 2018 Apr 5;19(3):452–9. doi: 10.5811/westjem.2018.2.36404 (PMC5942007; doi:10.5811/westjem.2018.2.36404)
Supplement: Supplementary file 2 [file wjem-19-452-s002.docx]

**Supplemental Table 2: Complete Case Analysis Multivariable Logistic Regression of Mortality Within 1^st^ 24 Hours**

|  | **Odds Ratio** | **95% CI** | **P** |
| --- | --- | --- | --- |
| **Age** | 1.0 | 0.93-1.07 | 0.90 |
| **Penetrating Injury** | 1.0 | 0.55-1.86 | 0.97 |
| **ED GCS** | 0.73 | 0.65-0.81 | <0.01 |
| **AIS Head** | 1.67 | 1.20-2.34 | <0.01 |
| **ED Intubation** | 0.30 | 0.15-0.57 | <0.01 |
| **Blood pressure** |  | | |
| **Hypotensive** | 2.86 | 1.26-6.50 | 0.01 |
| **Normotensive** | Reference | | |
| **95^th^-99^th^ Percentile** | 1.03 | 0.38-2.81 | 0.95 |
| **>99^th^ Percentile** | 2.70 | 1.38-5.28 | <0.01 |
