# Supplementary material for: Severely Elevated Blood Pressure and Early Mortality in Children with Traumatic Brain Injuries: The Neglected End of the Spectrum
Source: West J Emerg Med. 2018 Apr 5;19(3):452–9. doi: 10.5811/westjem.2018.2.36404 (PMC5942007; doi:10.5811/westjem.2018.2.36404)
Supplement: Supplementary file 3 [file wjem-19-452-s003.docx]

**Supplemental Table 3: Multivariable Logistic Regression of 24-Hour Mortality in Patients with Isolated Torso/ Abdominal Trauma**

|  | **Odds Ratio** | **95% CI** | **P** |
| --- | --- | --- | --- |
| **Age** | 0.90 | 0.81-1.01 | 0.09 |
| **Penetrating Injury** | 1.31 | 0.52-3.30 | 0.56 |
| **ED GCS** | 0.89 | 0.79-0.99 | 0.03 |
| **ISS** | 1.06 | 1.03-1.09 | <0.01 |
| **ED Intubation** | 0.62 | 0.19-2.04 | 0.43 |
| **Blood pressure** |  | | |
| **Hypotensive** | 5.48 | 2.11-14.22 | <0.01 |
| **Normotensive** | Reference | | |
| **95^th^-99^th^ Percentile** | 0.62 | 0.08-5.10 | 0.66 |
| **>99^th^ Percentile** | 0.94 | 0.24-3.68 | 0.92 |
