# Supplementary material for: Severely Elevated Blood Pressure and Early Mortality in Children with Traumatic Brain Injuries: The Neglected End of the Spectrum
Source: West J Emerg Med. 2018 Apr 5;19(3):452–9. doi: 10.5811/westjem.2018.2.36404 (PMC5942007; doi:10.5811/westjem.2018.2.36404)
Supplement: Supplementary file 4 [file wjem-19-452-s004.docx]

**Supplemental Table 4: Cox Regression of In-Hospital Mortality in Patients with Isolated Torso/ Abdominal Trauma**

|  | **Hazard Ratio** | **95% CI** | **P** |
| --- | --- | --- | --- |
| **Age** | 0.89 | 0.83-0.95 | <0.01 |
| **Penetrating** | 0.93 | 0.54-1.59 | 0.79 |
| **ED GCS** | 0.97 | 0.90-1.04 | 0.40 |
| **ISS** | 1.05 | 1.03-1.07 | <0.01 |
| **ED Intubation** | 1.53 | 0.72-3.28 | 0.27 |
| **Blood Pressure** |  | | |
| **Hypotensive** | 4.81 | 2.70-8.59 | <0.01 |
| **Normotensive** | Reference | | |
| **95^th^-99^th^ Percentile** | 1.10 | 0.41-2.92 | 0.85 |
| **>99^th^ Percentile** | 1.20 | 0.56-2.57 | 0.65 |
